# Intrinsic expression of host genes and intronic miRNAs in prostate carcinoma cells

**DOI:** 10.1186/1475-2867-9-21

**Published:** 2009-08-12

**Authors:** Kavleen Sikand, Stephen D Slane, Girish C Shukla

**Affiliations:** 1 Center for Gene Regulation in Health and Disease, Cleveland State University, 2121 Euclid Avenue, Cleveland, OH 44115, USA; 2 Department of Biological, Geological and Environmental Sciences, Cleveland State University, 2121 Euclid Avenue, Cleveland, OH 44115, USA; 3 Department of Psychology, Cleveland State University, 2121 Euclid Avenue, Cleveland, OH 44115, USA

## Abstract

**Background:**

Recent data show aberrant and altered expression of regulatory noncoding micro (mi) RNAs in prostate cancer (PCa). A large number of miRNAs are encoded in organized intronic clusters within many protein coding genes. While expression profiling studies of miRNAs are common place, little is known about the host gene and their resident miRNAs coordinated expression in PCa cells. Furthermore, whether expression of a subset of miRNAs is distinct in androgen-responsive and androgen-independent cells is not clear. Here we have examined the expression of mature miRNAs of miR 17–92, miR 106b-25 and miR 23b-24 clusters along with their host genes C13orf25, MCM7 and AMPO respectively in PCa cell lines.

**Results:**

The expression profiling of miRNAs and host genes was performed in androgen-sensitive MDA PCa 2b and LNCaP as well as in androgen-refractory PC-3 and DU 145 cell culture models of PCa. No significant correlation between the miRNA expression and the intrinsic hormone-responsive property of PCa cells was observed. Androgen-sensitive MDA PCa 2b cells exhibited the highest level of expression of most miRNAs studied in this report. We found significant expression variations between host genes and their resident miRNAs. The expressions of C13orf25 and miR 17–92 cluster as well as MCM7 and miR 106b-25 cluster did not reveal statistically significant correlation, thus suggesting that host genes and resident miRNAs may be expressed independent of each other.

**Conclusion:**

Our results suggest that miRNA expression profiles may not predict intrinsic hormone-sensitive environment of PCa cells. More importantly, our data indicate the possibility of additional novel mechanisms for intronic miRNA processing in PCa cells.

## Background

Prostate cancer (PCa) is the most frequently diagnosed cancer and the second leading cause of death among American men [1]. The initial stage of PCa is androgen-dependent; hence, androgen blockade is the mainstay of therapy. Although androgen ablation therapy is effective in causing regression of prostate tumors, it produces only temporary remissions. The therapy eventually fails and PCa transits to the androgen-refractory or androgen-independent stage, a lethal form with a fatal prognosis [2]. Although several mechanisms have been proposed for explaining the pathogenesis of PCa, the molecular mechanisms underlying the critical transition from androgen-dependence to androgen-independence of these tumors are poorly understood [reviewed in [3,4]].

Regulatory noncoding microRNAs (miRNAs) play a critical role in the regulation of gene expression [5] and are potential candidates for studying their role in the progression of PCa to androgen-independent stage. miRNAs control gene expression by binding to the complementary sites in the 3' untranslated regions (3' UTRs) of target mRNAs and triggering either translational inhibition or mRNA degradation by a molecular mechanism which is a subject of intense investigation [6-9]. Aberrant expression of miRNAs has been correlated with the metastatic potential of melanoma [10,11], lymphoma [12-15], gastric [16-18], ovarian [19-21], breast [22,23] and colorectal cancers [24-26]. Furthermore, miRNA expression or lack thereof, is associated with the disease progression of urogenital cancers including bladder, kidney [27,28] and prostate [29,30]. Recent studies have demonstrated the aberrant expression of miRNAs in PCa tissues and cell lines [30-34] and differential expression of a miRNA in androgen-dependent and androgen-independent PCa cells [29]. Furthermore, androgen-regulated miRNAs may play a role in the transition of PCa to the androgen-independent stage [35]. Therefore, the role of miRNAs and their differential signature expression patterns in androgen-dependent and androgen-independent PCa cells requires a closer validation.

Gene expression modulating miRNAs are encoded in diverse genomic locations including intergenic regions, introns of protein-coding genes and introns/exons of noncoding RNA genes [36]. Approximately one third of experimentally identified human miRNAs are encoded in the introns of annotated protein coding genes [37,38]. It is likely that the intronic miRNAs are processed from the same primary transcript as the precursor mRNAs and thus, their expression levels are regulated by the expression of the host mRNA [39]. Interestingly, over 40% of human miRNAs are organized in evolutionarily conserved clusters suggesting that the clustering propensity of miRNAs may have a significant biological role [36-38,40,41]. Accumulating evidence suggests that clustered miRNAs are transcribed as polycistronic transcripts and thus have similar levels of expression. The miRNA clusters located in the introns of host genes present an especially interesting scenario by adding another level of co-regulation with the host mRNA expression. Although several miRNAs including some clustered miRNAs are functionally involved in the process of carcinogenesis [42-44], the phenomenon of miRNA clustering and their expression analysis vis-à-vis host gene expression has not been extensively investigated in PCa cells.

We investigated the expression of intronic miRNA clusters with respect to their host transcript expressions in PCa cells representing two distinct stages of PCa. The panel of PCa cell lines included two androgen-sensitive (MDA PCa 2b and LNCaP) and two androgen-independent (PC-3 and DU 145) cell lines. MDA PCa 2b and LNCaP cell lines are derived from bone and lymph node metastasis respectively [45,46]. These cell lines express androgen receptor (AR) and PCa biomarker, prostate specific antigen (PSA) [47]. On the other hand PC-3 and DU 145 cell lines lack AR, do not express PSA and do not have androgen response [47]. Host genes and their resident miRNA clusters were selected based on their potential relevance to cancer. Three intronic miRNA clusters were analyzed in this study: (i) miR 17–92 cluster, (ii) miR 106b-25 cluster and (iii) miR 23b-24 cluster.

**Figure 1:**
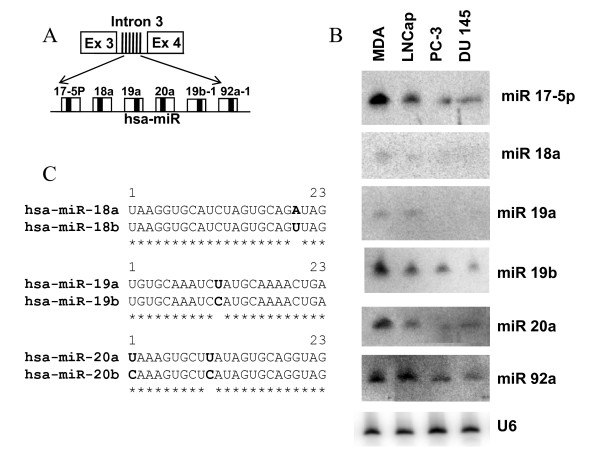
**miR 17–92 cluster structure and expression**. (A) Schematic representation of miR 17–92 cluster. Six miRNAs are expressed from intron 3 of C13orf25 gene (Ex = exon). The unfilled boxes correspond to precursor miRNAs and the solid thick black line within each box corresponds to mature miRNA. (B) The expression patterns of member miRNAs of miR 17–92 cluster in four PCa cell lines as evaluated by northern blotting (MDA = MDA PCa 2b). (C) Pairwise alignment of miRNAs. The figure reveals sequence similarities between miRNAs of paralogous clusters. Identical sequence between two miRNAs is shown by asterisks below the comparison and letters in bold correspond to sequence variation. miRs 18b and 20b shown in the alignments are derived from miR106a-363 cluster on chromosome X. The miR 106a-363 cluster contains six miRNAs, namely, 106a, 18b, 20b, 19b-2, 92a-2 and 363. The mature forms of miR 19b and miR 92a are expressed from both miR17–92 and miR106a-363 clusters.

**Figure 2:**
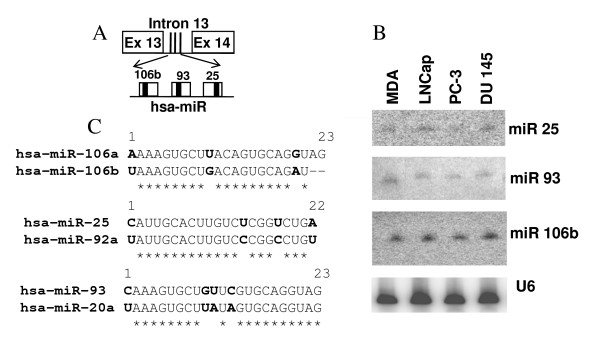
**miR106b-25 cluster structure and expression**. (A) Genomic organization of miR106b-25 cluster in intron 13 of MCM7 gene. Precursor miRNAs are represented as unfilled boxes and the solid thick black line within each box corresponds to mature miRNA (Ex = exon). (B) Representative northern blot showing the expression of miR 106b-25 cluster in PCa cell lines (MDA = MDA PCa 2b). (C) Sequence comparison of miRNAs. Identical sequence between two miRNAs is shown by asterisks below the comparison and letters in bold correspond to sequence variation. miR 106a is expressed from miR106a-363 cluster on chromosome X. miR 92a is derived from both miR17–92 and miR106a-363 clusters. miR 20a is also expressed from the miR 17–92 cluster.

**Figure 3:**
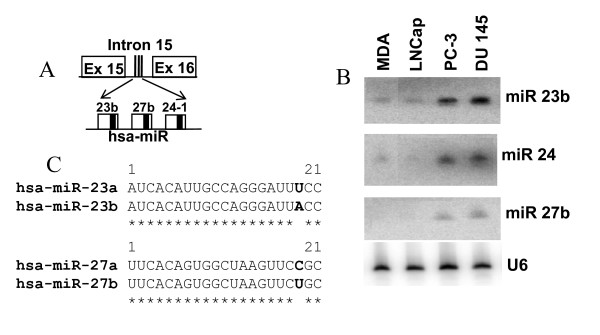
**miR23b-24 cluster structure and expression**. (A) Three miRNAs are encoded in intron 15 of AMPO gene (Ex = exon). Precursor miRNAs are represented as unfilled boxes and the solid thick black line within each box corresponds to mature miRNA. (B) Representative northern blot showing the expression of miR 23b-24 in PCa cell lines (MDA = MDA PCa 2b). (C) Aligned sequences of mature miRNAs revealing the high level of identity. Asterisks correspond to identical sequence between two miRNAs and bold letters corresponds to sequence variation. miRs 23a and 27a are expressed from the miR 23a-24 cluster on chromosome 19.

The human miR 17–92 cluster is comprised of six miRNAs and is located in the third intron of C13orf25 gene at 13q31.3 (Figure 1A). A growing body of evidence indicates that the miR 17–92 cluster acts as a potential oncogene and is mechanistically involved in tumorigenesis [48-50]. The miR 106b-25 cluster consists of three miRNAs and is located in the 13^th ^intron of MCM7 (minichromosome maintenance protein 7) gene on human chromosome 7 (Figure 2A). The miRNAs of this cluster share a high degree of sequence homology with the miRNAs of the miR17–92 cluster, thus suggesting the possibility of overlapping cellular functions of the two miRNA clusters. Furthermore, overexpression of MCM7 has been associated with PCa progression and recurrence [51]. In addition, miR 106b-25 is believed to be involved in a variety of cancers, suggesting its biological significance in carcinogenesis [16]. The miR 23b-24 cluster resides in intron 15 of the Aminopeptidase O (AMPO) gene on human chromosome 9 and consists of three member miRNAs: miR 23b, miR 24 and miR 27b (Figure 3A). The aberrant expression of miRNA members of miR 23b-24 cluster has been reported in some cancers [27]. miR 24 appears to inhibit erythroid differentiation [52] and a role for miR 27b has been indicated in angiogenesis [53]. The differential expression of these miRNA clusters has not been studied systematically in prostate carcinogenesis. Furthermore, whether the member miRNAs are differentially expressed in androgen-dependent and androgen-refractory PCa is undetermined.

We hypothesized that these miRNAs may have unique expression patterns in androgen-sensitive and androgen-refractory cell culture models of PCa. Hence, we characterized the expression of the miRNAs and their host genes in PCa cell lines by northern blot and real-time quantitative PCR techniques.

## Results

### Expression Profiling of miR 17–92, 106b-25 and 23b-24 Clusters in Androgen-Dependent and Androgen-Refractory PCa Cell Lines by Northern Blotting

Two distinct stages of PCa are recapitulated in a variety of human PCa cell culture models. These cells lines are characterized in terms of their androgen sensitivity to account for their cell invasion and metastasis properties. As shown in Figures 1 to 3, we characterized the expression of miRNA members of three clusters in PCa cell lines by Northern blotting. A summary of the relative miRNA expression is summarized in table 1. The oncomir cluster 17–92 is encoded in the third intron of the C13orf25 gene (Figure 1A) [12]. As evidenced by the northern blotting experiment, miRNA members of the miR 17–92 cluster show a characteristic differential expression profile for each cell line studied (Figure 1B). All miRNA members of this cluster were expressed in androgen-sensitive MDA PCa 2b and LNCaP cells, albeit with varied levels (Figure 1B). However, not all the members were expressed in hormonal-refractory PC-3 and DU 145 cell lines e.g. miR18a and miR19a expression was not detected by northern blot analysis in these hormonal-refractory PCa cell lines. miRs19b, 17-5p, 20a and 92a were found to be expressed in all four cell lines, albeit the expression was at lower levels in hormonal-refractory cells as compared to hormone-sensitive cells (Figure 1B). The miR 17–92 cluster members on chromosome 13 share a high level of sequence homology with the miRNAs of two paralogous clusters, namely, miR 106b-25 and miR 106a-363 present on chromosome 7 and chromosome X respectively. Sequence homology between the miRNAs of these clusters is shown in Figures 1C and 2C.

**Table 1:** Relative expression of miRNAs as determined by northern blots

Cluster	Genomic Location	Host Gene	hsa-miR	MDA PCa 2b	LNCaP	PC3	DU145
**17–92**	13q31.3	C13orf25	17-5p	++++	+++	++	++
			18a	++	+	-	-
			19a	++	+	-	-
			19b	++++	+++	+	+
			20a	+++	++	+	+
			92a	+++	+++	++	++
							
**106b-25**	7q22.1	MCM7	25	+	+	+	+
			93	+	+	+	+
			106b	++	++	++	++
							
**23b-24**	9q22.32	AMPO	23b	+	+	+++	+++
			24	+	+	++	++
			27b	-	-	+	+

Hybridization signal intensities of each miRNA probe were normalized to the hybridization signal intensities of the U6 snRNA probe and the relative expression was scored on an arbitrary scale where (-) and (++++) correspond respectively to undetectable and highest levels of relative expression of each miRNA in the four PCa cell lines.

The miR 106b-25 cluster is encoded in intron 13 of MCM7 gene and contains three miRNA coding genes. The organization of miRNA genes in intron 13 is shown in Figure 2A. The northern blot analyses of miR 106b-25 cluster revealed similar levels of expression of all three miRNAs in the four cell lines (Figure 2B).

The miR 23b-24 cluster contains three miRNA coding genes located in the intron 15 of AMPO gene. The structure of miR 23b-24 cluster is shown in Figure 3A. The miRNA members of miR 23b-24 cluster demonstrated varied degree of expression levels as observed by signal intensities (Figure 3B). All three member miRNAs are expressed at higher levels in androgen-independent PC-3 and DU 145 cell lines as compared to androgen-dependent cells. The miR 23b-24 cluster on chromosome 9 shares sequence homology with miR 23a-24 cluster on chromosome 19. The miR 23b and 27b sequences (miR 23b-24 cluster) differ by one nucleotide from the miR 23a and 27a sequences in the miR 23a-24 cluster (Figure 3C) and the two miRs 24 arising from both the clusters have an identical sequence. Hence, it is possible that the signal intensities of miRNA expression levels observed by northern blotting may correspond to some degree of cross hybridization to the paralog counterparts of miRNAs.

In summary, northern blot analyses of miRNA clusters in PCa cell lines demonstrated differential expression patterns of 12 miRNAs in the four PCa cell lines. Although it is a reliable technique, northern blotting cannot accurately estimate the expression of miRNAs having highly similar sequences. Hence, we sought to quantify the expression of these miRNAs as well as their host transcripts by reverse transcription and quantitative real-time PCR. To quantify the expression of mature miRNAs, we used TaqMan MicroRNA assays [54,55]. This technique can accurately analyze the expression of related miRNAs because it can discriminate between similar miRNAs differing by a single nucleotide [56].

### Expression of miR 17–92 Cluster and Host Transcript C13orf25

**Figure 4:**
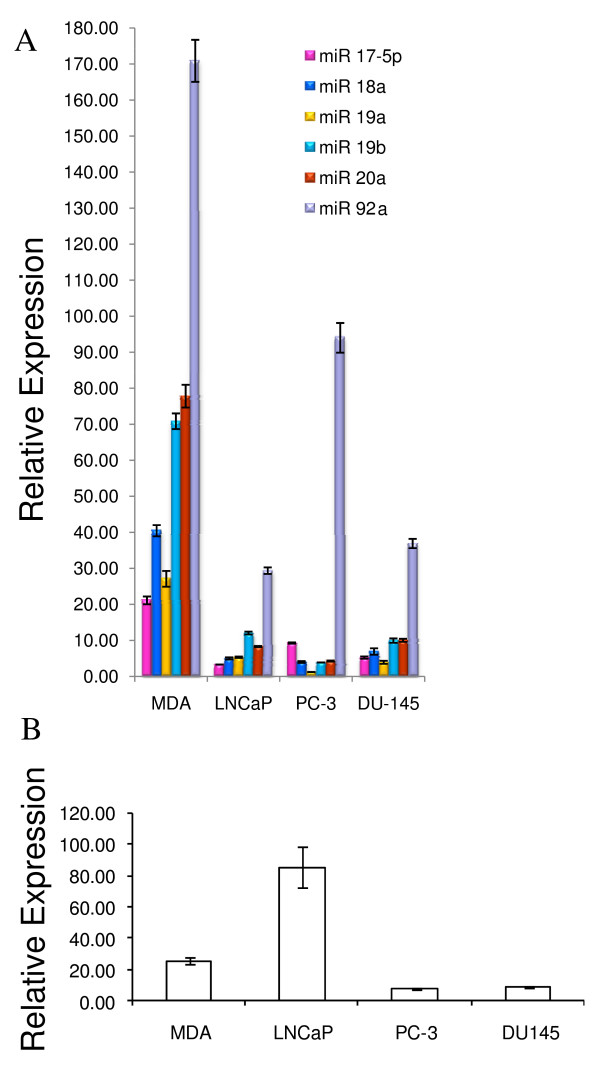
**Quantitative real-time PCR expression profile of miR 17–92 cluster and the host transcript C13orf25 in prostate cancer cell lines**. (A) miRNA expression as determined by qRT-PCR. The relative expression of each miRNA is plotted as 2^-ΔCt ^after normalization to sno66 expression. Each bar represents mean ± SEM of at least 3 replicates. (B) qRT-PCR analysis of C13orf25 mRNA expression. Each bar represents C13orf25 mRNA expression normalized to GAPDH mRNA expression. Data are plotted as mean ± SEM of six replicates. (MDA = MDA PCa 2b).

All six miRNA members of the miR 17–92 cluster were significantly downregulated (p < 0.01, table 2 & Figure 4A) in LNCaP, PC-3 and DU 145 cells as compared to their expression in MDA PCa 2b cells. The expression profile of the miR 17–92 cluster in LNCaP (androgen-dependent) cells was similar to that in DU 145 (androgen-independent) cells instead of matching the miR 17–92 expression profile in androgen-dependent MDA PCa 2b cells (Figure 4A). The maximum downregulation (upto 20 fold) of miRNA members was observed in PC-3 (androgen-independent) cells and this expression profile was significantly different from that in the other three cell lines (table 2). This suggests that the miRNAs of the miR 17–92 cluster are differentially expressed among the four PCa cell lines and do not correlate with the intrinsic androgen response status of PCa cells. Remarkably, although there is a general decrease in the expression of miRNA members in the three cell lines as compared to their expression in MDA PCa 2b cells, the fold decrease differs widely among cluster members. While the expression of miR 17-5p and miR 92a in PC-3 cells is reduced to approximately half their levels in MDA PCa 2b cells, the miRs 18a, 19a, 19b and 20a are downregulated by 10–20 fold in PC-3 cells as compared to their expression in MDA PCa 2b cells (Figure 4A). Likewise, the member miRNAs in LNCaP and DU 145 cells are downregulated in the range of 4–9 folds as compared to their expression in MDA PCa 2b cells. Within each cell line, the member miRNAs are expressed at different levels with miR 92a being the most abundant in all cell lines. It is noteworthy that miR 19b and miR 92a are also derived from a paralogous cluster miR 106a-363 on chromosome X. Hence, the levels of miRs 19b and 92a estimated by qRT-PCR do not represent the amount of these miRNAs processed from the miR 17–92 cluster alone but instead, represent the total amount of these miRNAs derived from both clusters.

**Table 2:** Fold changes in the expressions of C13orf25 mRNA and miR 17–92 cluster in PCa cell lines

	LNCaP/MDA	PC-3/MDA	DU145/MDA	PC-3/LNCaP	DU145/LNCaP	DU145/PC-3
C13orf25	3.35**	0.29***	0.34****	0.08**	0.10**	1.16
miR 17-5p	0.15**	0.43**	0.24***	2.79****	1.57*	0.56***
miR 18a	0.12****	0.09****	0.17****	0.80	1.38	1.72*
miR 19a	0.19**	0.04**	0.14***	0.23**	0.73*	3.16*
miR 19b	0.17****	0.05****	0.14****	0.32**	0.82*	2.55***
miR 20a	0.10****	0.05****	0.12****	0.51****	1.21*	2.35***
miR 92a	0.17***	0.55***	0.21***	3.20****	1.25**	0.39***

The fold change in the expression levels of the host gene and member miRNAs was determined by ratios of the expression between two cell lines (LNCaP/MDA, PC-3/MDA, DU145/MDA, PC-3/LNCaP, DU145/LNCaP and DU145/PC-3). Asterisks indicate statistical significance as determined using the independent samples t-test.

*p < 0.05, **p < 0.01, ***p < 0.001, ****p < 0.0001

As seen in Figures 4A and 4B, the expression profile of C13orf25 transcript in PCa cell lines is distinguishable from the expression profile of the resident miR 17–92 cluster in the same cell lines. While the C13orf25 mRNA is approximately 3-fold upregulated (p < 0.01, table 2) in LNCaP cells as compared to its expression in MDA PCa 2b, PC-3 and DU 145 cells, the member miRNAs of the miR 17–92 cluster are downregulated in LNCaP cells as compared to their expression in MDA PCa 2b cells (Figures 4A &4B). We applied Pearson product-moment correlation test on the C13orf25 and miR 17–92 expression data and found no significant correlation between the expression of the host gene C13orf25 and its resident miRNAs (table 3).

**Table 3:** Pearson product-moment correlations of C13orf25 mRNA expression with the expression of resident miRNAs.

		miR17-5p	miR18a	miR19a	miR19b	miR 20a	miR 92a
C13orf25	Pearson correlation coefficient	-0.343	-0.126	-0.004	-0.036	-0.098	-0.366
	Significance (1-tailed)	0.329	0.437	0.498	0.482	0.451	0.317

*Correlation is significant at 0.01 level

### Expression of miR 106b-25 Cluster and Host Transcript MCM7

**Figure 5:**
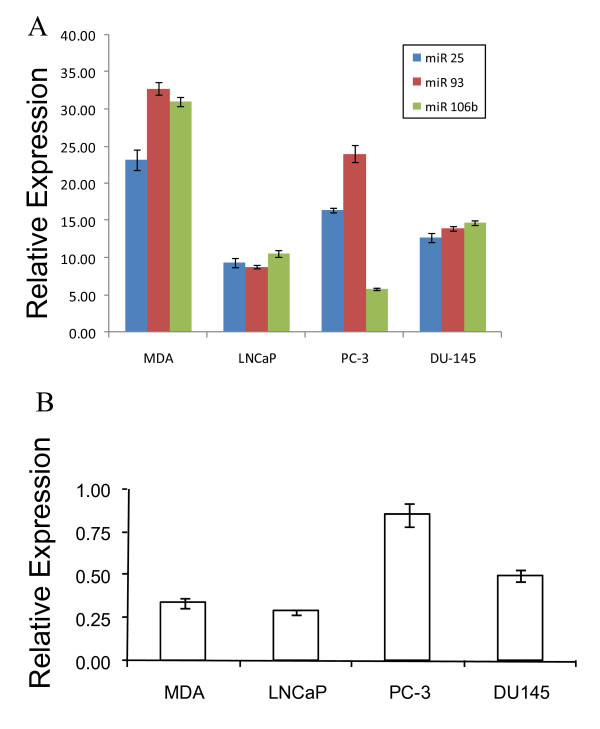
**Expression profiles of member miRNAs of miR 106b-25 cluster and the host gene MCM7 in prostate cancer cell lines**. (A) miRNA expression as determined by qRT-PCR. The relative expression of each miRNA is plotted as 2^-ΔCt ^after normalization to sno66 expression. Each bar represents mean ± SEM of at least 3 replicates. (B) qRT-PCR analysis of MCM7 mRNA expression. Each bar represents MCM7 mRNA expression normalized to GAPDH mRNA expression. Data are plotted as mean ± SEM of six replicates. (MDA = MDA PCa 2b).

Next, we analyzed the expression of three miRNAs, namely miR 25, miR 93 and miR 106b, which are members of the miR 106b-25 cluster encoded in intron 13 of MCM 7 gene. The expression of the three miRNA members of the miR 106b-25 cluster was significantly reduced (p < 0.01, table 4) in LNCaP, PC-3 and DU 145 cells as compared to their expression in MDA PCa 2b cells (Figure 5A). While all the three member miRNAs were downregulated by more than 2-fold in LNCaP and DU 145 cells, miR 106b was downregulated by approximately 5-fold in PC-3 cells. miR 25 and miR 93 showed less than 2-fold downregulation in PC-3 cells as compared to their expression in MDA PCa 2b cells (Figure 5A). The 106b-25 miRNA cluster expression profile in androgen-dependent LNCaP cells was significantly different (p < 0.001, table 4) from that in another androgen-dependent cell line MDA PCa 2b. Moreover, the miRNA expression profile in PC-3 (androgen-independent) cells was also significantly different (p < 0.01, table 4) from that in DU 145 (androgen-independent) cells. Hence, the expression of miR 106b-25 cluster does not appear to correlate with the intrinsic androgen responsiveness of PCa cells.

**Table 4:** Fold changes in MCM7 mRNA and miR 106b-25 expressions in PCa cell lines.

	LNCaP/MDA	PC-3/MDA	DU145/MDA	PC-3/LNCaP	DU145/LNCaP	DU145/PC-3
MCM7	0.84	2.52****	1.46**	2.97***	1.72***	0.58***
miR 25	0.40***	0.70**	0.55**	1.75***	1.35*	0.77**
miR 93	0.26****	0.73**	0.42****	2.72***	1.58***	0.58***
miR 106b	0.34****	0.18****	0.47****	0.55***	1.38**	2.50****

The fold change in the expression levels of mRNA and miRNAs was determined by ratios of the expression between two cell lines (LNCaP/MDA, PC-3/MDA, DU145/MDA, PC-3/LNCaP, DU145/LNCaP and DU145/PC-3). Asterisks indicate statistical significance as determined using the independent samples t-test. *p < 0.05, **p < 0.01, ***p < 0.001, ****p < 0.0001

The comparison of the expression profile of MCM7 mRNA with the expression profile of the resident miR 106b-25 cluster shows no significant correlation between the host gene and intronic miRNA cluster expression (table 5). While the member miRNAs were significantly downregulated in LNCaP, PC-3 and DU 145 cells as compared to their expression in MDA PCa 2b cells (Figure 5A), the MCM7 mRNA was significantly upregulated in DU 145 cells (p < 0.01, table 4) and PC-3 cells (p < 0.0001, table 4), and was expressed at similar level in LNCaP cells as compared to the expression in MDA PCa 2b cells (Figure 5B).

**Table 5:** Pearson product-moment correlations of MCM7 mRNA expression with the expression of resident miRNAs.

		miR 25	miR 93	miR 106b
MCM 7	Pearson correlation coefficient	0.089	0.216	-0.582
	Significance (1-tailed)	0.455	0.392	0.209

*Correlation is significant at 0.01 level

### Expression of miR 23b-24 Cluster and Host Transcript AMPO

**Figure 6:**
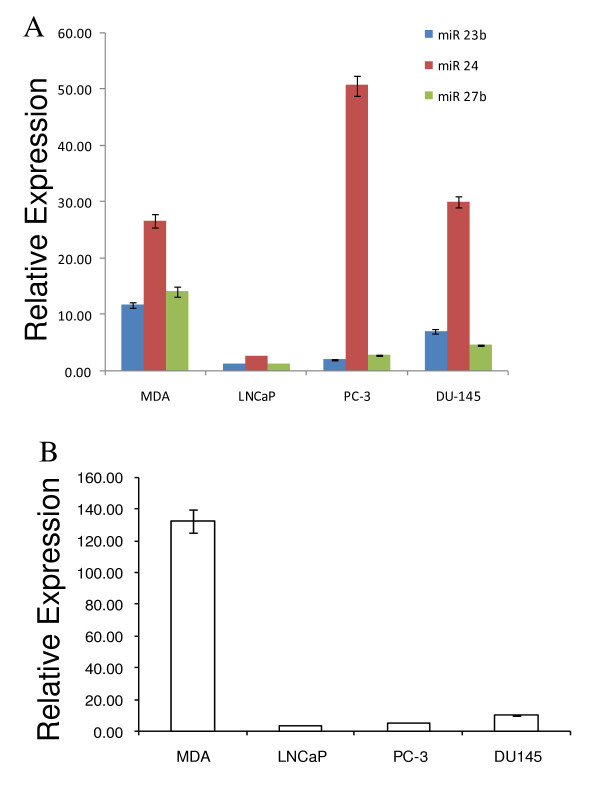
**Differential expression of miR 23b-24 cluster and the host gene AMPO in prostate cancer cell lines**. (A) miRNA expression as determined by qRT-PCR. The relative expression of each miRNA is plotted as 2^-ΔCt ^after normalization to sno66 expression. Each bar represents mean ± SEM of at least 3 replicates. (B) qRT-PCR analysis of AMPO mRNA expression. Each bar represents AMPO mRNA expression normalized to GAPDH mRNA expression. Data are plotted as mean ± SEM of six replicates. (MDA = MDA PCa 2b).

All three members of miR 23b-24 cluster, namely miR 23b, miR 24 and miR 27b were significantly downregulated by approximately 10 fold (p < 0.01, table 6) in LNCaP cells as compared to their expression in MDA PCa 2b cells (Figure 6A). While miR 23b and miR 27b were significantly downregulated in PC-3 and DU 145 cells, miR 24 was upregulated by about 2-fold in PC-3 cells and was expressed at almost the same level in DU 145 cells as compared to the expression in MDA PCa 2b cells (Figure 6A, table 6). The miRNA expression profile is significantly different (p < 0.01, table 6) in the four PCa cell lines, thus suggesting that miR 23b-24 cluster expression is not indicative of the intrinsic androgen responsive status of PCa cells. In all four PCa cell lines, miR 24 is the most abundantly expressed member miRNA. The mature miR 24 is also derived from a paralogous miR 23a-24 cluster on chromosome 19 comprising of miR 23a, miR 27a and miR 24. Hence, the level of miR 24 observed here presumably represents the total miR 24 expression derived from both paralogous clusters.

**Table 6:** Fold changes in the expressions of the host transcript AMPO and the resident miR 23b-24 cluster in PCa cell lines.

	LNCaP/MDA	PC-3/MDA	DU145/MDA	PC-3/LNCaP	DU145/LNCaP	DU145/PC-3
AMPO	0.02****	0.03****	0.07****	1.69****	3.24****	1.91***
miR 23b	0.09****	0.17****	0.60****	1.83****	6.29****	3.43****
miR 24	0.10****	1.90****	1.12	18.95****	11.20****	0.59****
miR 27b	0.07**	0.19**	0.33**	2.52**	4.23***	1.67***

The fold change in the expression levels of the host gene and member miRNAs was determined by ratios of the expression between two cell lines (LNCaP/MDA, PC-3/MDA, DU145/MDA, PC-3/LNCaP, DU145/LNCaP and DU145/PC-3). Asterisks indicate statistical significance as determined using the independent samples t-test. *p < 0.05, **p < 0.01, ***p < 0.001, ****p < 0.0001.

The expression of AMPO transcript was reduced by approximately 13 fold in LNCaP, PC-3 and DU 145 cells as compared to its expression in MDA PCa 2b cells (Figure 6B, table 6). This expression pattern matches the downregulation of all three member miRNAs of the miR 23b-24 cluster in LNCaP cells and the lower expression of miRs 23b and 27b in PC-3 and DU 145 cells as compared to the expression in MDA PCa 2b cells (Figure 6A). Pearson correlation analysis of the AMPO and miR 23b-24 expression data suggests a positive correlation between the expression of AMPO gene and miRs 23b and 27b but no significant correlation between the expression of AMPO and miR 24 (table 7). However, since the expression of miR 24 observed here could potentially represent the total miR 24 expression derived from miR 23b-24 cluster (residing in AMPO gene on chromosome 9) as well as its paralogous cluster miR 23a-24 (chromosome 19), the miR 24 expression may be disregarded in the interpretation of correlation with the host gene expression.

**Table 7:** Pearson product-moment correlations of AMPO mRNA expression with the expression of resident miRNAs.

		miR 23b	miR 24	miR 27b
AMPO	Pearson correlation coefficient	0.871	-0.013	0.978*
	Significance (1-tailed)	0.065	0.494	0.011

*Correlation is significant at 0.05 level

## Discussion

The objectives of this study were: (1) to determine if a subset of miRNAs has a specific expression pattern in androgen-sensitive and androgen-independent PCa cell lines; (2) if there is a correlation in the expression levels of miRNAs and their host genes; and finally (3) to statistically examine if the expression of these miRNAs can delineate the intrinsic hormonal-sensitivity of the PCa cells studied here.

Several miRNA expression profiling studies have identified unique, differential miRNA expression patterns in different cancer types leading to the successful use of miRNA signatures for the molecular classification of cancer [57,58]. Hence, it was pertinent to search for miRNA expression profiles unique to androgen-dependent and androgen-independent PCa cells. However, whether miRNA expression profiles can predict the intrinsic androgen sensitivity of PCa cells is still an open question. Only a few studies so far have addressed the correlation, if any, between miRNA expression and intrinsic androgen responsive status of PCa cells. In this context, we investigated the expression of 12 miRNAs organized in three clusters (miR 17–92, miR 106b-25 and miR 23b-24) in two androgen-dependent (MDA PCa 2b and LNCaP) and two androgen-independent (PC-3 and DU 145) PCa cell lines using northern blotting and qRT-PCR analysis.

Northern blot analysis pointed out unique differences between the miRNAs of a given cluster. Some miRNAs appear to have unique expression in androgen-dependent and androgen-independent PCa cells (Figures 1, 2, 3, table 1). However, since probes used for northern blotting cannot distinguish between closely related miRNA sequences, we relied on the analysis of data generated by qRT-PCR. For all three miRNA clusters, member miRNAs are most abundantly expressed in MDA PCa 2b cells and are downregulated in LNCaP, PC-3 and DU 145 cell lines. In the miR 17–92 cluster, the expression profile of all the six member miRNAs in androgen-dependent MDA PCa 2b cells is significantly distinguishable from that in another androgen-dependent cell line, LNCaP (Figure 4A & table 2). Likewise, the expression profiles of miR 17–92 cluster members in PC-3 and DU 145 cells, both androgen-independent PCa cell types, significantly differ from each other (Figure 4A & table 2). This suggests that the expression of the miR 17–92 cluster does not correlate with the intrinsic androgen-responsive status of PCa cells. Similarly, in the miR 106b-25 cluster (Figure 5A, table 4) and in the miR 23b-24 cluster (Figure 6A, table 6), the expression profiles of member miRNAs do not match in either androgen-dependent cell lines (MDA PCa 2b and LNCaP) or two androgen-independent cell lines (PC-3 and DU 145).

None of the host genes studied here appears to contain consensus androgen responsive elements (ARE) and thus may not be potential direct targets of AR mediated transcriptional activation. To further confirm if host genes or miRNAs may respond to noncanonical androgen signaling, we treated LNCaP cells growing in charcoal stripped serum with dihydrotestosterone (DHT) and measured the expression of host genes and several miRNAs by qRT-PCR. The comparison of the expression profiles in untreated and DHT treated LNCaP cells did not demonstrate any statistically significant variation (data not shown), suggesting that the expression of host genes and miRNA clusters is not influenced by hormone signaling.

Although with the set of miRNAs studied here, a unique miRNA signature for androgen response status of PCa cells was not identified, a few investigations have reported the differential expression of miRNAs in androgen-dependent and androgen-independent PCa cells. A recent study suggested the potential of miRNA expression profiles to classify the clinical PCa samples according to their androgen dependence [30]. Further, Galardi et al. [33] reported that miR 221 and miR 222 are overexpressed in PC-3 cellular model of aggressive PCa as compared with LNCaP and 22R*v*1 cell line models of slow growing carcinomas. These miRNAs were found to contribute to PCa pathogenesis by targeting the tumor suppressor p27^kip1^. Most recently, miR 146a and miR 125b were reported to be differentially expressed in androgen-dependent PCa cells as compared to their expression in androgen- independent PCa cells [29,35]. Further investigations evaluating a large number of miRNAs are required to fully address the question of potential association between miRNA expression and intrinsic androgen sensitivity of PCa cells.

The expression of the miRNA clusters examined in this study has not been previously evaluated in PCa cells. Only one report has shown the overexpression of miR 17-5p, miR 20a (miR 17–92 cluster) and miR 25 (miR 106b-25 cluster) in prostate tumor samples [31]. With regard to the expression of miR 23b-24 cluster in PCa, the downregulation of miR 23b and miR 27b in clinical PCa samples as compared to their expression in benign prostatic hyperplasia samples has been reported [30]. In the present study, we observed intriguing differences in miRNA expression across the four PCa cell lines. The most striking observation in our data is the magnitude of fold differences in the expression of miRNA cluster members across the four cell lines. The member miRNAs of the miR 17–92 cluster are downregulated by a range of 5 to 9 fold in LNCaP cells as compared to their expression in MDA PCa 2b cells; in PC-3 cells, miRs 18a, 19a, 19b and 20a are downregulated by 10–20 fold as compared to their expression in MDA PCa 2b cells and in DU 145 cells, downregulation of member miRNAs ranges from 4 to 8 fold (Figure 4A & table 2). Likewise, the member miRNAs of miR 106b-25 cluster are downregulated by 2–5 fold in LNCaP, PC-3 and DU 145 cells as compared to their expression in MDA PCa 2b cells (Figure 5A & table 4). In the miR 23b-24 cluster, the miR 24 expression measured by qRT-PCR does not estimate the miR 24 expression derived from miR 23b-24 cluster alone; instead, it represents the combined miR 24 expression from miR 23b-24 cluster and its paralog miR 23a-24 cluster present on chromosome 19. This could explain the high expression of miR 24 as compared to the expression of miRs 23b and 27b observed in our study. There is a highly significant downregulation (1.5 to 12 fold) of miRs 23b and 27b in LNCaP, PC-3 and DU 145 cell lines as compared to MDA PCa 2b cell line (Figure 6A & table 6). It is reasonable to speculate that such a magnitude of fold change in the expression of miRNAs across the four PCa cell lines may have significant biological consequences. It is possible that these clustered miRNAs may be playing important roles in the pathogenesis of PCa. In this context, some target mRNAs of the miR 17–92 cluster members have been experimentally validated. These targets include both tumor suppressors (p21, Bim, PTEN, Rb2/p130, Rbl2) as well as oncogenes (E2F1, AIB1) suggesting that the miRNAs of the miR 17–92 cluster can act as both oncogenes or tumor suppressors by modulating cell proliferation in a cell-type-specific manner depending on the set of target mRNAs that are expressed [42,49,59-62]. The experimentally validated targets of miR 106b-25 cluster also include the tumor suppressors, Bim and p21 as well as the oncogene, E2F1 [44,16]. Likewise, several mRNA targets of miR 23b-24 cluster, namely CYP1B1 and hALK4 have been identified [52,63,64]. It would be interesting to evaluate the expression of these targets in PCa cell culture models and clinical samples.

A few studies have compared the expression profiles of miRNAs and their host genes. Significant correlation between miRNA and host gene expression was reported suggesting the coregulation of intronic miRNA and host gene expression [65,66]. In this study, we profiled both the miRNA and host gene expression in four PCa cell lines. A positive correlation was observed between the expression of AMPO transcript and its resident miRs 23b and 27b (table 7). No significant correlation was observed between the expression of C13orf25 transcript and the expression of its resident miR 17–92 cluster members (table 3). Likewise the comparison of MCM7 mRNA expression with the expression of its resident miR 106b-25 cluster members shows no significant correlation (table 5). This appears to be in contrast to a previous finding which observed a perfect correlation between MCM7 mRNA expression and miR 106b-25 precursor miRNA expression [16]. Interestingly, the levels of miR 106b-25 precursor miRNAs do not appear to correlate perfectly with the levels of the mature forms of these miRNAs [16]. This observation is consistent with our findings as we have quantified the mature forms of miRNAs in the study.

Although we have shown the absence of correlation between the miRNA and the host transcript expression in two out of the three miRNA clusters, it may be a widespread phenomenon. Contrary to previous observations, our results suggest that host gene expression may not be a reliable indicator of the intronic miRNA expression. Our findings raise interesting questions regarding the biogenesis of intronic miRNAs. Since the expression of intronic miRNAs does not correlate with the expression of the host transcript, could it be that instead of being derived from the spliced intron of the host transcript, the resident miRNAs are transcribed independently from an alternative promoter in response to intracellular signaling? Another possibility could be that the resident miRNAs are co-transcribed with the host gene but additional, yet unknown, regulatory events occurring during the post-transcriptional processing of primary miRNAs to mature miRNAs may lead to diverse miRNA expression as compared to the host gene expression.

## Conclusion

In conclusion, we studied the expression of 12 miRNAs from three clusters and compared their expression with the host gene expression in androgen-sensitive and androgen-refractory PCa cell lines. Our data indicate that the expression of none of these 12 miRNAs correlates with the androgen-dependent and androgen-refractory stages of the PCa cell culture models. MDA PCa 2b cells were unique in terms of overexpression of most of the miRNAs studied here. More importantly, in two out of the three miRNA clusters, we found no correlation between the host gene expression and resident miRNA expression, thus suggesting that the expression of some host genes and their resident miRNAs may be independent of each other. Our findings indicate the possibility of novel regulatory mechanisms for the processing of intronic miRNAs. Whether these mechanisms are cancer-specific is an open question. Further investigations directed to evaluate the complex phenomenon of intronic miRNA biogenesis in cancer cells may lead to the identification of new therapeutic targets.

## Methods

### Cell Lines and Cell Culture

The androgen-dependent human PCa cell lines MDA PCa 2b and LNCaP, and androgen-independent PCa cell lines PC-3 and DU145 were obtained from American Type Culture Collection (Manassas, VA). MDA PCa 2b cells were cultured in BRFF-HPC1 medium (Athena Enzyme Systems, Baltimore, MD) supplemented with 20% fetal bovine serum (FBS) and antibiotics (100 units/ml of penicillin G sodium; 100 μg/ml streptomycin sulphate). LNCaP, PC-3 and DU145 cells were cultured in RPMI 1640 medium supplemented with 10% FBS, 2 mM L-glutamine and antibiotics. All cell lines were maintained in a humidified 5% CO_2 _atmosphere at 37°C.

### RNA Extraction

Total RNA was isolated from 80% confluent cells using Trizol reagent (Invitrogen, Carlsbad, CA) according to manufacturer's instructions. RNA yield and purity were determined spectrophotometrically at 260–280 nm and the integrity of RNA verified by electrophoresis through denaturing agarose gels stained with ethidium bromide.

### Northern Blotting

Total RNA samples (30 μg each) were separated on 15% polyacrylamide gels containing 7 M urea and electroblotted to nylon membranes (Hybond-XL, Amersham Biosciences). After UV cross-linking the RNA to nylon membrane at 120 mJ for 1 minute, the membrane was prehybridized in 10 ml ULTRAhyb-Oligo hybridization buffer (Ambion) at 35°C for 30 minutes and then, hybridized in the same buffer with γ-^32^P labelled probes at 35°C overnight. DNA oligonucleotides complementary to mature miRNAs and U6 small nuclear RNA (snRNA) were used as probes. Probes were 5' end-labelled using [γ-^32^P] ATP and T4 polynucleotide kinase (Promega, Madison, WI). After overnight hybridization, the membrane was washed twice with 6× SSC for 15 minutes and 0.1% SDS with 6× SSC for 10 minutes, each at room temperature and then exposed to phosphor screen overnight. Bands were visualized by scanning the screens on Typhoon 9410 Variable Mode Imager (Amersham Biosciences). Expression of U6 snRNA was used as a loading control. Blots were stripped by incubating in 1% SDS at 65°C for 1 hour and reprobed.

### Reverse Transcription and Quantitative Real-Time (qRT) PCR Analysis of Mature miRNA Expression

First strand cDNA was synthesized from 10 ng of total RNA using primers specific for mature miRNAs and small nucleolar RNA 66 (sno66). The miRNA-specific primers were obtained from TaqMan MicroRNA Assays (Applied Biosystems Foster City, CA). Reagents for cDNA synthesis were obtained from TaqMan MicroRNA Reverse Transcription kit (Applied Biosystems). For each sample, a 15 μl reverse transcription (RT) reaction was set up containing 10 ng of total RNA, 1× RT buffer, 1 mM of dNTP mix, 50 units of MultiScribe reverse trancriptase, 3.8 units of RNase inhibitor and 3 μl of miRNA-specific RT primer. The reactions were incubated in a thermal cycler (BIORAD PTC-100) at 16°C for 30 minutes, 42°C for 30 minutes, 85°C for 5 minutes and then held at 4°C. The 'reverse transcriptase minus' controls were also synthesized under the same conditions. In order to quantify the mature miRNAs and sno66 in each sample, the cDNAs were amplified using TaqMan MicroRNA Assays together with the TaqMan 2× Universal PCR Master Mix (Applied Biosystems Foster City, CA). Briefly, a 20 μl reaction was set up containing 1.33 μl product from RT reaction, 1 μl of 20× TaqMan microRNA assay mix (mixture of miRNA-specific forward and reverse primers, and miRNA-specific TaqMan MGB probe labeled with FAM fluorescent dye) and 10 μl of TaqMan 2× Universal PCR Master Mix. These reactions were dispensed into a 96-well optical plate and the plate was positioned in 7500 Real-time PCR System (Applied Biosystems) under the following conditions: 95°C for 10 minutes followed by 40 cycles of 95°C for 15 seconds and 60°C for 1 minute. Three replicates were performed per RT reaction together with the 'reverse transcriptase minus' and 'no template' controls. Duplicate PCRs were performed for all miRNAs in each RNA sample. The mean C_t _was determined from the replicates. Sno66 expression was used as an invariant control. The relative expression of each miRNA was calculated as 2^-ΔCt ^where ΔCt = Ct value of each miRNA in a sample – Ct value of sno66 in that sample. All experiments were repeated at least twice with three replicates and two independent RNA samples. All replicates provided similar results.

### Quantitative Real-Time PCR Analysis of Host Gene Expression

Total RNA was isolated from the cells as described above. To remove DNA contamination from the RNA samples, 10 μg of total RNA was incubated with 10 units of DNase I (Amplification grade; Invitrogen, Carlsbad, CA) at 37°C for 30 minutes. 1 μg of DNase I treated RNA was reverse transcribed into cDNA using the ImProm-II Reverse Transcription System (Promega, Madison, WI). Briefly, a 20 μl RT reaction was set up containing 1× ImProm-II reaction buffer, 5.5 mM MgCl_2_, 0.5 mM of each dNTP, 20 units of RNasin ribonuclease inhibitor, 0.5 μg of oligo (dT_15_) primer, 1 μl of ImProm-II reverse transcriptase and 1 μg of RNA. The RT reactions were incubated at 25°C for 5 minutes followed by 42°C for 1 hour and reverse transcriptase was inactivated by heating at 70°C for 15 minutes. Primers for real-time PCR were designed using the Primer Express software (v3.0; Applied Biosystems). The SYBR Green ER qPCR SuperMix Universal was obtained from Invitrogen and used for setting up real-time PCR reactions. In brief, a 20 μl reaction was set up containing 1× SYBR Green Supermix (Invitrogen), 0.05 μM of each of the forward and reverse primers, 50 nM ROX dye and 2 μl of template cDNA. The reactions were dispensed into 96-well optical plates and the amplification was carried out in 7500 Real-time PCR System (Applied Biosystems) under the following conditions: 50°C for 2 minutes, 95°C for 10 minutes followed by 40 cycles of 95°C for 15 seconds and 60°C for 1 minute. Three replicates were performed per cDNA sample along with the 'reverse transcriptase minus' and 'no template' controls. Each PCR with three replicates was repeated twice. The specificity of amplification was confirmed by melting curve analysis and also by running the PCR reactions on 3% agarose gels. Gene expression was quantified using the relative standard curve method. Different dilutions of cDNA synthesized from HeLa cells were used to plot the standard curves for each gene. Glyceraldehyde-3-phosphate dehydrogenase (GAPDH) expression was used as an internal control and the relative expression of each gene was normalized to GAPDH expression. Mean normalized expression of each gene ± SE was calculated from the PCR replicates. All experiments were repeated at least twice with three replicates and two independent RNA samples and similar results were obtained. Following primers were used for the host gene expression analysis: (i) C13orf25 Forward ACACGCGGCCAGGTTAAG, Reverse CGCTGCAAAGATCGTTTGAG; (ii) MCM7 Forward CATGAGGCGTTACATAGCCATGT, Reverse CTCTCGCCTCATCTCCACGTA; (iii) AMPO Forward GGCCATGGGTGTGTACCTCTA, Reverse GACCTATCCATCTGCTCCTTGGT; (iv) GAPDH Forward ACCCACTCCTCCACCTTTGAC, Reverse TGTTGCTGTAGCCAAATTCGTT.

### Statistical Analysis

The statistical analysis of expression levels of 12 miRNAs and three host precursor mRNAs and their coexpression in PCa cell lines was performed utilizing the SPSS package. The index of expression of each miRNA was 2^-ΔCt ^after normalization to sno66 expression levels. The following analyses were conducted:

(i) Pairwise comparison of miRNAs selected from three clusters (17–92, 106b-25, 23b-24) across four PCa cell lines using independent samples t-test.

(ii) Pairwise comparison of the three selected host genes across four PCa cell lines using independent samples t-test.

(iii) The degree of linearly related expression levels between the host gene and resident miRNAs was determined by Pearson product-moment correlation coefficients.

## List of abbreviations

(PCa): Prostate Cancer; (AR): Androgen Receptor; (PSA): Prostate Specific Antigen; (AMPO): Aminopeptidase O; (UTR): Untranslated Region; (MCM): Mini Chromosome Maintenance; (ORF): Open Reading Frame; (miRNA): MicroRNA.

## Competing interests

The authors declare that they have no competing interests.

## Authors' contributions

KS performed all the experiments. KS and GCS designed and analyzed the data. KS, SDS and GCS performed the statistical analysis. KS and GCS interpreted the statistical data and wrote the manuscript.
